# The Application of Enhanced Depth Imaging Spectral-Domain Optical Coherence Tomography in Macular Diseases

**DOI:** 10.1155/2020/9503795

**Published:** 2020-08-24

**Authors:** Jing Wang, Lian-Rong Yin

**Affiliations:** Department of Refractive Surgery, Eye Hospital, China Academy of Chinese Medical Sciences, Beijing 100040, China

## Abstract

The choroid plays an essential role in the pathogenesis of various posterior segment diseases. However, traditional imaging methods still have limited cross-sectional observation of choroid. Enhanced depth imaging in spectral-domain optical coherence tomography (EDI SD-OCT) uses a closer scanning position to the eye to create an inverted SD-OCT image with the advantage of better depth sensitivity, which can observe choroidal structure and measure choroidal thickness (CT) accurately. At present, more and more choroidal thickness measurements have been made in normal and pathologic states, in order to understand the pathogenesis and differential diagnosis and prognosis of various diseases, especially for macular lesions. This paper would review relevant original literatures published from January 1, 2008, to February 1, 2020, to evaluate the relationship between the changes of CT with EDI SD-OCT and macular diseases.

## 1. Introduction

The choroid is an integral structure in the eye that accounts for most of the ocular blood flow and contributes blood supply to the prelaminar portion of the optic nerve and outer retina. [1]. Therefore, it is very critical to accurately measure the changes of the choroid thickness for many posterior segment diseases. However, contact B-scan ultrasonography can only detect information of thickening choroid in disease state, and indocyanine green angiography is useful for detecting choroidal vascular structure, but neither of them can accurately provide choroidal cross section information [2].

OCT is a noninvasive, noncontact, transpupillary imaging modality that has recently been introduced into clinical practice. It uses light waves to obtain high-resolution cross-sectional images of the retina, creating an in vivo “optical biopsy” of the macular area. EDI-OCT system places the objective lens of the SD-OCT closer to the eye, so that the light backscattered from the choroid is closer to the zero delay line and the sensitivity of these low signal areas can be enhanced and provided inverted images of retina on the lower choroid. Compared with traditional OCT, this image can display choroidal structure more clearly and help to accurately evaluate the cross-sectional structure and thickness of choroid [1–3].

Especially with the development of en face OCT imaging and optical coherence tomography angiography, the choroid could be assessed in a layer-by-layer manner from choriocapillaris to choroidal large vessel layer, and retinal and choroidal quantified vessel density and blood flow could be generated [4, 5]. These methods are helpful to observe the pathophysiological characteristics of various macular diseases, such as age-related macular degeneration, polypoidal choroidal vasculopathy, and central serous chorioretinopathy. This article discusses the clinical application of EDI-OCT in macular diseases.

## 2. Choroidal Thickness

Before the advent of EDI SD-OCT, choroidal thickness was obtained by autopsy without data in vivo. According to histological findings of autopsy, the mean choroidal thickness was between 170 *μ*m and 220 *μ*m [1]. The choroid is a highly vascular structure with blood-flow and thickness varying in relation to the intraocular pressure, perfusion pressure, endogenous nitric oxide production, vasoactive, secretory production of choroidal ganglion cells, endogenous circulating catecholamines, and its intrinsic vasomotricity. After death, all these functions fail or cease to exist. Fixation of tissue before histological analysis causes shrinkage, thus affecting any measured thicknesses. As such, histological analysis can only provide a rough estimate, but not an accurate measurement, of the thickness of the choroid in vivo.

In 2008, Spaide et al. [1] reported the measurement of subfoveal choroidal thickness (SFCT) in 17 normal subjects with an average age of 33.4 years by EDI SD-OCT. The results showed that the SFCT was 318 *μ*m in the right eye and was 335 *μ*min the left eye, and there was a high correlation between the two eyes. Later, Margolis et al. [2] reported SFCT was 287 ± 76 *μ*m (mean ± SD) with a sample size of 30 subjects (54 eyes) using the Spectralis, and Manjunath et al. [6] reported SFCT was 272 ± 81 *μ*m with a sample size of 34 normal subjects (34 eyes) using the Cirrus HD-OCT. Ikuno et al. [7] measured the choroidal thickness of 43 healthy Japanese with an average age of 39.4 years by using 1060 nm OCT and similar results were obtained. Recently, Wei et al. [8] reported the mean SFCT was 253.8 ± 107.4 *μ*m with a relatively large sample size of 3233 subjects. At the same time, the choroidal thickness is different in different positions of the posterior part of the eye [2, 9]. The subfoveal choroid is the thickest, thicker on the temporal side than on the nasal side and on the superior side than on the inferior side. Angiographic studies have also confirmed that the subfoveal choroid is thickest, which demonstrated that the choroidal arteries and choriocapillaris in the macular area fill more rapidly and densely than elsewhere in the retina. As the densest part of blood vessels in the whole body, the change of choroid thickness is often closely related to the change of blood vessel density. Many factors in the body can lead to the loss of choroidal vessels, such as age, system, or eye diseases. The loss of microvasculature may reduce the ability of choroid to provide adequate oxygen and other metabolites to RPE and outer retina.

It was found that there is a negative correlation between choroidal thicknesses and age. Regression analysis showed that the SFCT decreased 2∼4 *μ*m for each year of age [8, 10]. This analysis implies that, over the course of an 80-year lifetime, an eye would lose approximately one-third of its subfoveal choroidal thickness. However, some studies have found that the SFCT of individuals who are under 60 years old is not related to age, while over 60 years of age, choroidal thickness decreases about 4∼5.4 *μ*m every year with the increase of age [8, 11, 12].

In addition, the researchers also found that the SFCT was correlated negatively with the axial length and refractive error. In highly myopic eyes (greater than 6D), the SFCT decreased by 12.7 *μ*m for each decade of life and 8.7 *μ*m for each D of myopia [9]. Another study also reported that the estimation of the variation in the subfoveal choroidal thickness in relationship to the axial length was −43.84 *μ*m/mm. In the myopic group, the variation in the subfoveal choroidal thickness with the myopic refractive error was −10.45 *μ*m per diopter [13].

Zeng et al. [14] reported using EDI SD-OCT to measure choroidal thickness in normal subjects. It was found that the choroidal thickness of men was higher than that of women in all age groups. Gender was also one of the factors affecting choroidal thickness. Later, Tuncer et al. [15] demonstrated that the choroidal thickness of men was more than that of women, and the average thickness of men was 99.16 *μ*m compared to that of women. Ooto et al. [16] also reported that the average thickness of men was about 62.2 *μ*m as compared to that of women. The possible causes were analyzed: human estrogen receptor subtype mRNA gene, gender, and hormone level may affect choroidal blood flow and thus affecting choroidal thickness.

Recent studies have also reported that the choroidal thickness was related to race and smoking. Rhodes et al. [17] used EDI SD-OCT to measure the peripapillary choroidal thickness of a group of normal people of African and European origin. The results showed that the peripapillary choroidal thickness of normal people of African origin is thicker than that of normal people of European origin. However, EDI SD-OCT was performed on 90 randomly selected healthy individuals of White, Black, and South Asian origin. Bafiq et al. [10] reported that there was no difference in the subfoveal choroidal thickness between ethnic groups but the temporal choroid was significantly thinner in Black subjects. Mean macular choroidal thickness was thinner for smokers (148 ± 63 *μ*m) than for nonsmokers (181 ± 65 *μ*m) among all diagnostic categories. Subgroup analysis of patients with AMD features revealed a similar reduced choroidal thickness in smokers (121 ± 41 *μ*m) compared with nonsmokers (146 ± 46 *μ*m). Chronic cigarette smoke exposure may be associated with reduced choroidal thickness [18].

## 3. Application of Pathogenesis in Macular Diseases

Age-related macular degeneration (AMD) is the most common cause of blindness in both developed countries and developing countries in people older than 60 years [19, 20]. Compared with Asians and Africans, Europeans had a higher prevalence of early and any age-related macular degeneration [21]. The pathophysiological changes of AMD are complicated. In addition to genetics, lipofuscin formation, drusen formation, local inflammation, and neovascularization are all the pathogenic factors. In AMD patients, retinal pigment epithelial detachment (PED) is not uncommon, and its mechanism is controversial. Gass [22] thought that PED formation could be attributed to the growth of neovascularization through the Bruch membrane and into the sub-RPE space with concurrent extravasation of fluid or blood. Bird and Marshal [23] proposed that the interaction between the PRE and the Bruch membrane was crucial to RPE detachment, while choroidal neovascularization (CNV) is the result of PED. Later, some researchers proposed that retinal vascular proliferation without choroidal vascular involvement is also a common cause of PED, which is called retinal hemangioma proliferation [24].

Fundus fluorescein angiography (FFA) mostly shows late hyperfluorescence within the PED and PEDs usually appear optically in OCT. Because commercially available OCT instruments have limited ability to image down to and include the choroid under the PED, they are unable to distinguish if deeper portions within the PED are filled with fluid without reflectivity or just are not imaged in the first place. The emergence of EDI SD-OCT breaks through this limitation to a certain extent, and this technology can clearly show the internal structure of PED. In 22 eyes with PED secondary to exudative AMD, using EDI-OCT, Spaide [25] found that the entire PED cavity was filled with hyperreflective tissue in 11 eyes. In the other 11 eyes, what appeared to be serous fluid and collections of reflective material was seen to be contiguous with subretinal pigment epithelial neovascularization, had angiography suggestive of fibrovascular proliferations, and was seen to course up along the back surface of the RPE. The presence of any of these materials could be expected according to the Gass hypothesis of PED formation [22]. With the development of OCT technology, we can use this technology to segment and quantify RPE elevation. PED subtypes are generally divided into drusenoid PEDs, serous PEDs, vascularized PEDs, and mix PEDs [26]. Recently, several studies indicated that drusenoid PEDs could be a recognized phenotypic manifestation of AMD, and serous PEDs were more common in CSC and PCV, less in neovascular AMD [27, 28].

Polypoidal choroidal vasculopathy (PCV) is a peculiar disorder involving the macula and is characterized by multiple, recurrent serosanguineous detachment of the retinal pigment epithelium (RPE) and neurosensory retina secondary to leakage and bleeding from choroidal vascular lesions [29]. PCV is more common in the Asians than in Caucasians [21, 30]. Until now, the precise pathogenesis of PCV is still unknown, and whether it is a variant of neovascular AMD or a distinct clinical entity remains controversial. At present, there are few studies on the pathology of PCV. Shiraga et al. [31] carried out histological examination on the abnormal tissue stripped by surgery and found that the abnormal vascular membrane was the fibrovascular tissue between choroid and retina. Okubo et al. [32] found that there were more dilated venules and arterioles in the inner layer of the choroid; therefore, it was considered that the polypoid lesions were caused by the dilation of the choroidal venules. Several studies using EDI-OCT have shown that the choroid thickness of PCV patients was significantly more than that of normal patients, and the choroid thickness of healthy eyes in monocular PCV patients was also significantly larger [33, 34]. However, Lee et al. reported that the eyes with normal or subnormal SFCT exhibited thickening of extrafoveal choroid at sites of polypoidal disease; unlike other pachychoroid diseases, PCV have a wide range of choroidal thickness and the choroidal thickness does not always thicken in PCV [35].

Central serous chorioretinopathy (CSC) is a disease characterized by an exudative detachment of the neurosensory retina, often in association with serous RPE detachment [36, 37]. At present, the pathogenesis of CSC is still unclear, but it is generally believed that the primary lesions of CSC are RPE and choroidal capillaries, and discoid retinal detachment is the secondary lesion. A large number of studies have shown that the choroid of CSC patients is in a high circulation state. ICGA also shows multiple leakage, congestion, and venous dilation of choroidal vessels. Donald and Gass [38] speculated that the increased permeability of the choriocapillaris resulted in pigment epithelial detachment and fluid exuded into the subretinal space. It was found that there were multifocal areas of choroidal vascular hyperpermeability in CSC patients during ICGA. Using EDI-OCT, Imamura et al. [39] reported that the mean choroidal thickness in 28 eyes of 19 patients with CSC was 505 (±124) *μ*m, which was 214 *μ*m (85%) greater than the mean choroidal thickness of age-matched normal eyes. In addition, two studies reported both eyes of patients with unilateral CSC and observed that increased choroidal thickness was present in both eyes: the affected eyes (445.58 ± 100.25 *μ*m) and the unaffected fellow eyes (378.35 ± 117.44 *μ*m) compared with normal eyes (266.80 ± 55.45 *μ*m) [40, 41]. Although the pathophysiology of CSC remains unclear, these findings provide additional evidence that CSC may be caused by increased choroidal hydrostatic pressure. Later, Kuroda et al. [42] demonstrated that increased choroidal vascular area was observed in the whole macula area in eyes with CSC. That finding suggested that CSC may originate from a choroidal circulatory disturbance.

Idiopathic macular hole (IMH) is a full-thickness deficiency of retinal tissue involving the anatomic fovea and affecting central visual acuity, which occurs spontaneously in the absence of ocular trauma, inflammation, ametropia, ocular vascular diseases, and other primary diseases. At present, the pathogenesis of IMH is widely accepted by Gass's theory of vitreofoveal traction [43, 44]. However, some patients with IMH were found to have no obvious posterior vitreous detachment. In recent years, the results showed that the abnormal choroidal blood circulation was related to some age-related macular lesions and the thickness of choroid in patients with IMH was different from that in normal eyes, but this result was controversial. Reibaldi et al. [45] compared the SFCT in 22 eyes with full-thickness idiopathic macular hole with unaffected fellow eyes and healthy controls. They reported the mean SFCT of 183.2 *μ*m in eyes with macular hole group, 196.6 *μ*m in the fellow-eye group, and 245.0 *μ*m in the control group, suggesting that bilateral thinning of the choroid may precede a macular hole. There was no correlation between subfoveal choroidal thickness and the diameter of the hole.

## 4. Application of Differential Diagnosis in Macular Diseases

Both PCV and exudative AMD feature abnormal vascular lesions arising from the choroidal vessels, which lead to recurrent serous exudation and hemorrhages. Some studies suggested that the two diseases may share certain common environmental risk factors and genetic factors [30]. Some similar clinical features make it difficult to distinguish PCV from exudative AMD, but their prognosis and response to treatment are different [46]. Compared with exudative AMD, PCV has a slower progression and better visual prognosis, better therapeutic response to photodynamic therapy (PDT), and less responsiveness to antivascular endothelial growth factors (VEGF) therapy [47]. The current results show that choroidal thickness measurement has high reliability and repeatability [48]; therefore, the assessment of choroidal thickness is helpful to distinguish exudative AMD from PCV. Using EDI SD-OCT, Lee et al. [35] compared SFCT in eyes with PCV (25 eyes of 21 patients) with exudative AMD (30 eyes of 30 patients) and early AMD (17 eyes of 16 patients). The mean SFCT in eyes with PCV and in uninvolved fellow eyes was 438.3 ± 87.8 *μ*m and 372.9 ± 112.0 *μ*m, respectively, which was significantly greater than in eyes of age-matched normal subjects (224.8 ± 52.9 *μ*m), in eyes with exudative AMD (171.2 ± 38.5 *μ*m), and in eyes with early AMD (177.4 ± 49.7 *μ*m). The results showed that the subfoveal choroidal thickness of PCV was significantly thicker than that of AMD. Spaide et al. [36] also showed the same results in eyes with neovascular AMD (21 eyes of 21 patients) and in eyes with polypoidal choroidal vasculopathy (PCV) (23 eyes of 23 patients). Additionally, it was found that eyes with the subfoveal choroidal thickness of ≥300 *μ*m are more than 5 times more likely to have PCV. Similar results were also observed in another study that compared the choroidal thickness among eyes with neovascular AMD, PCV, and CSC. It was demonstrated that the choroid was thicker in eyes with PCV and CSC than in normal subjects or in those with neovascular AMD [49]. These findings suggested the involvement of different pathogenic mechanisms between PCV and exudative AMD. Researchers believe that the thicker choroid in PCV patients could be partially attributed to the dilation of middle and large choroidal vessels or an increase in the choroidal vascular permeability [50]. It not only was choroidal neovascularization with angiocystic dilation of capillaries at the border, but also may be significantly different from AMD in choroidal structure, which indicates that PCV and exudative AMD may have different pathogenesis [36].

There are some similarities in choroidal changes between PCV and CSC, and both choroidal thicknesses are significantly larger than in normal subjects or in those with neovascular AMD [39]. Kim et al. [49] reported that the choroid was thicker in PCV (319.92 ± 68.66 *μ*m) and CSC patients (367.81 ± 105.56 *μ*m) than in controls (241.97 ± 66.37 *μ*m) and AMD patients (186.62 ± 64.02 *μ*m). It has been suggested that there may be similar pathogenesis between the two diseases. The thicker choroid in PCV patients may be caused by the dilation of middle and large choroidal vessels or an increase in the choroidal vascular permeability. Although the typical CSC and PCV manifestations are easy to distinguish, in some cases, some persistent or recurrent CSCs may be difficult to distinguish from PCV. The isolated macular lesions of PCV may have similar clinical and angiographic features to CSC. Toyama et al. [51] reported that, compared with the typical AMD group and control group, the PCV group had more history of CSC, while the Asians were more likely to have PCV and CSC than the Caucasians. Ahuja et al. also suggested that PCV may be a sequela of chronic CSC, and the two diseases may have some common pathogenesis [52]. Using en face swept-source OCT, Baek et al. [53] showed that there were similarities in vascular density of the large choroidal vessel layer and pachyvessel pattern between CSC and thick-choroid PCV and between NV-AMD and thin-choroid PCV, which suggested that these three diseases may have common pathophysiological mechanism, involving choroidal changes.

## 5. Evaluation of Therapeutic Effect in Macular Diseases

It is considered that CSC may resolve spontaneously, and treatment may be required in eyes with persistent leakage. In fact, in the long-term serous retinal detachment of macula, it is impossible for the normal directional physiological chimerism between the optic extracellular segment and the villous process of RPE cells to return to normal. The longer the course of disease is, the less perfect the chimerism is and the less perfect the recovery of visual function is. Deng et al. [54]also reported that the visual prognosis of CSC patients was closely related to the height of macular retinal detachment. Currently, clinical studies have shown that laser photocoagulation (LP) is used in classic CSC, but is ineffective in chronic CSC, while PDT has a significant effect on both, and the recurrence rate is low [55, 56]. Maruko et al. [57] measured the SFCT before and after the treatment in 20 eyes of 20 patients with chronic CSC by SD-OCT. In the LP group (12 eyes), the mean choroidal thickness was 345 ± 127 *μ*m at baseline and 340 ± 124 *μ*m at 4 weeks after treatment, with no significant difference. The mean choroidal thickness in the PDT group (8eyes) was 389 ± 106 *μ*m at baseline and 330 ± 103 *μ*m at 4 weeks after treatment, with a significant decrease. ICGA also showed decreased hyperpermeability in the PDT group after treatment. This indicates that LP cannot change the choroidal thickness, but the choroidal thickness decreases significantly after PDT, which implies that the mechanism of LP and PDT is different. ICGA showed that the hyperpermeability state of choroid still existed after laser photocoagulation, and the thickness of choroid did not change after treatment. Therefore, it was considered that laser photocoagulation may change the environment around the leakage point, but it has no significant effect on choroidal function. Later, Pryds and Larsen [58] observed the SFCT of 16 CSC patients before and after PDT; Maruko et al. [59] treated CSC patients with half-dose verteporfin PDT and followed up for a year to observe the SFCT. It was all found that the SFCT decreased significantly after PDT. These findings suggest that PDT may lead to changes in choroidal function in CSC patients, reduce choroidal hyperpermeability, and reduce choroidal vascular leakage. Therefore, choroidal thickness may be used as an additional parameter to assist in the differentiation of CSC from other causes of serous retinal detachment. In addition, it can also be used as an evaluation of the efficacy of PDT and a follow-up observation index.

Currently, there is no very effective method to treat PCV. No matter PDT alone, anti-VEGF alone, or PDT combined with anti-VEGF treatment, most of them are partially relieved symptoms. The polypoid lesion can not be completely eliminated or easily relapsed, and all kinds of treatment methods have their special side effects. EVERST study [60] was a double-blind, multicenter study to evaluate the efficacy of PDT combined with ranibizumab or PDT alone versus ranibizumab monotherapy in patients with symptomatic macular polypoidal choroidal vasculopathy. The results of 6 months showed that PDT combined with ranibizumab or PDT alone was superior to ranibizumab monotherapy in achieving complete polyp regression. Using EDI SD-OCT, Maruko et al. [61] observe the SFCT of patients with PCV treated with PDT alone or in combination with anti-VEGF. After PDT alone and PDT combined with anti-VEGF, it was found that the polypoid lesion in all eyes were alleviated within 3 months after treatment, the SFCT was decreased compared with that before treatment, and the thickness of retina in combined treatment group was lower than that in PDT group after 6 months. In addition, the authors suggested that the choroidal thickening seen in PCV eyes was associated with increased choroidal vascular hyperpermeability. Therefore, PDT may not only lead to polypoid lesions atrophy, but also reduce the permeability of choroid. Recently, Elabban et al. [62] discussed the changes in choroidal thickness with ranibizumab treatment for PCV and exudative AMD. The results showed that the mean choroidal thickness showed no significant change at 1 month after the loading phase or at final examination. It is suggested that the effect of ranibizumab on choroidal thickness is smaller than expected. However, Ting et al. reported that the subfoveal choroidal thickness decreased significantly in both typical AMD and PCV eyes; all patients received anti-VEGF therapy over 12 months [63].

At present, there is a consensus on the standardized treatment of neovascular AMD, that is, the load period of antivascular therapy by intravitreal injection once a month for the first three months and the maintenance period of repeated treatment according to the follow-up results. However, multiple intraocular injection increases the risk of treatment, and patients will bear a huge financial burden. Recently, it has been found that stereotactic radiotherapy (SRT) can reduce the number of repetitive intravitreal injections (IVIs) in patients with neovascular AMD as a new adjuvant treatment. Ranjbar et al. [64] used EDI-OCT to measure SFCT and central macular thickness (CMT) in patients with neovascular AMD who received SRT and one mandatory IVI and followed up for at least 12 months. The results showed that the annual injection rate decreased from 6.86 before SRT to 3.46 afterward, and the SFCT and CMT decreased 37 *μ*m and 71 *μ*m, respectively, compared with baseline at 12-month follow-up. The authors found that eyes with a thicker baseline SFCT needed fewer IVIs after SRT. Baseline SFCT may be helpful in predicting which patients with neovascular AMD will respond more favorably to SRT. Another study [65] also pointed out that the SFCT may be a predictive factor for visual outcome and treatment response in typical exudative AMD after intravitreal ranibizumab injections. The results show that the responder group had thicker subfoveal choroid (257.2 ± 108.3 *μ*m) and smaller lesions (1.3 ± 0.8 *μ*m) at baseline than the nonresponder group (167.1 ± 62.4 *μ*m and 2.0 ± 1.0 *μ*m). The responder group showed significantly better best-corrected visual acuity (BCVA) and thicker subfoveal choroid than the nonresponder group at 302 months and 602 months.

In conclusion, with people's understanding of eye diseases, the value of choroids is getting more and more attention. The emergence of EDI SD-OCT technology provides a noninvasive, intuitive, and repeatable tool for observing choroidal characteristics and chorioretinal diseases. It can accurately measure the parameters of fundus in vivo and has important clinical value in evaluating the changes of fundus parameters of various fundus diseases, especially macular diseases. However, there are few studies on this technology, which are limited to normal people and limited diseases, and the sample size is small. At present, EDI SD-OCT technology requires manual measurement of choroid thickness, which reduces the accuracy and comparability of the results. Perhaps this is also an important reason why there are fewer research projects using this software. If the software can automatically measure the thickness of the same site, its accuracy will be greatly improved, making our conclusions more scientific and objective. EDI SD-OCT technology must also be applied to more disease research.
